# BRCAness as a Biomarker of Susceptibility to PARP Inhibitors in Glioblastoma Multiforme

**DOI:** 10.3390/biom11081188

**Published:** 2021-08-11

**Authors:** Mary-Ann Xavier, Fernando Rezende, Ricardo Titze-de-Almeida, Bart Cornelissen

**Affiliations:** 1 Central Institute of Sciences, Technology for Gene Therapy Laboratory, University of Brasília—UnB/FAV, Brasília 70910-900, Brazil; fernando@veterinario.med.br (F.R.); ricardotitze.unb@gmail.com (R.T.-d.-A.); 2 Department of Oncology, Radiobiology Research Institute, University of Oxford, Oxford OX3 7LJ, UK; bart.cornelissen@oncology.ox.ac.uk; 3 Department of Nuclear Medicine and Molecular Imaging, University of Groningen, 9700 RB Groningen, The Netherlands

**Keywords:** glioblastoma, PARP, PARP inhibitors, BRCAness

## Abstract

Glioblastoma multiforme (GBM) is the most common primary brain cancer. GBMs commonly acquire resistance to standard-of-care therapies. Among the novel means to sensitize GBM to DNA-damaging therapies, a promising strategy is to combine them with inhibitors of the DNA damage repair (DDR) machinery, such as inhibitors for poly(ADP-ribose) polymerase (PARP). PARP inhibitors (PARPis) have already shown efficacy and have received regulatory approval for breast, ovarian, prostate, and pancreatic cancer treatment. In these cancer types, after PARPi administration, patients carrying specific mutations in the breast cancer 1 (BRCA1) and 2 (BRCA2) suppressor genes have shown better response when compared to wild-type carriers. Mutated BRCA genes are infrequent in GBM tumors, but their cells can carry other genetic alterations that lead to the same phenotype collectively referred to as ‘BRCAness’. The most promising biomarkers of BRCAness in GBM are related to isocitrate dehydrogenases 1 and 2 (IDH1/2), epidermal growth factor receptor (EGFR), phosphatase and tensin homolog (PTEN), MYC proto-oncogene, and estrogen receptors beta (ERβ). BRCAness status identified by accurate biomarkers can ultimately predict responsiveness to PARPi therapy, thereby allowing patient selection for personalized treatment. This review discusses potential biomarkers of BRCAness for a ‘precision medicine’ of GBM patients.

## 1. Introduction

Glioblastoma multiforme (GBM) has a radically altered genome and epigenome [1,2], which partially explains its aggressiveness. These modifications comprise point mutations, changes in the gene copy number, complete rearrangements, and epigenetic alterations [3]. Part of these genetic aberrations arise from the dysregulation of one or more molecular pathways responsible for recognizing and repairing DNA damage. Collectively, this molecular network is dubbed the DNA damage repair (DDR) machinery. When damaged DNA cannot be repaired, a functional DDR machinery triggers a signaling cascade, leading to cell senescence or apoptosis. These signaling pathways are disrupted in GBM, and despite the accumulation of DNA damage, cancerous cells will thrive, maintaining survival and pathological cell division [2,4].

While genomic instability contributes to the poor prognosis for GBM patients [5], the damaged DNA offers a target for pharmacological approaches that induce cancer cell death by a mechanism called synthetic lethality. This killing process occurs only if two molecular pathways are simultaneously deficient in one cell, whereas the isolated defect is innocuous [6]. One class of drugs that explores this killing mechanism are the inhibitors of poly(ADP-ribose) polymerase (PARP) enzyme, an important DDR component. PARP inhibitors (PARPis) can induce synthetic lethality in cancer cells with preexistent defects in the homologous recombination (HR) repair pathway, such as deleterious mutations of the breast cancer 1 (BRCA1) and breast cancer 2 (BRCA2) suppressor genes [7,8].

The majority of GBM clinical trials evaluating PARPis do not investigate the BRCA status to compare outcomes between patient groups [9]. One possible explanation is the expected low frequency of BRCA mutations in GBM, compared to breast, ovarian, and pancreatic cancer [10,11]. However, the reduction of BRCA expression due to the modulation of EF2 transcriptional factors, cyclin-dependent kinases changes in methylation of histones [12], or disruption of other DDR effectors [13] can similarly impair HR repair, resulting in the same phenotype observed for BRCA mutations. Collectively, these alterations that mimic BRCA mutations are known as ‘BRCAness’ [14]. Unraveling of GBM biology found mutations [1] in other genes that also result in BRCAness and confer sensitivity to PARPi treatment.

The increased understanding of GBM molecular landscape [1,15,16] has revealed promising biomarkers for prognostic assessment, resistance mechanisms [4,15], and targets for new therapies. We examine here the value of biomarkers that indicate the BRCAness status in GBM as an objective decision-making criterion for adding PARPi in the current glioma therapy.

## 2. PARPi and Synthetic Lethality

PARP is a family of enzymes that comprises 17 members with different functions, such as maintenance of genomic stability, transcriptional regulation, and cell death [16,17]. PARP1 is the best-characterized member of this family and plays a pleiotropic role in DDR, thus becoming an attractive target for cancer therapy [18]. The major role of PARP1 is binding to and repairing DNA insults, mainly single-strand breaks (SSBs) through the base excision repair (BER) pathway [19]. PARP1 uses NAD+ as a substrate to modify acceptor proteins with poly(ADP-ribose) (PAR) and forms a scaffold, which in turn attracts BER components [16,17].

**Figure 1:**
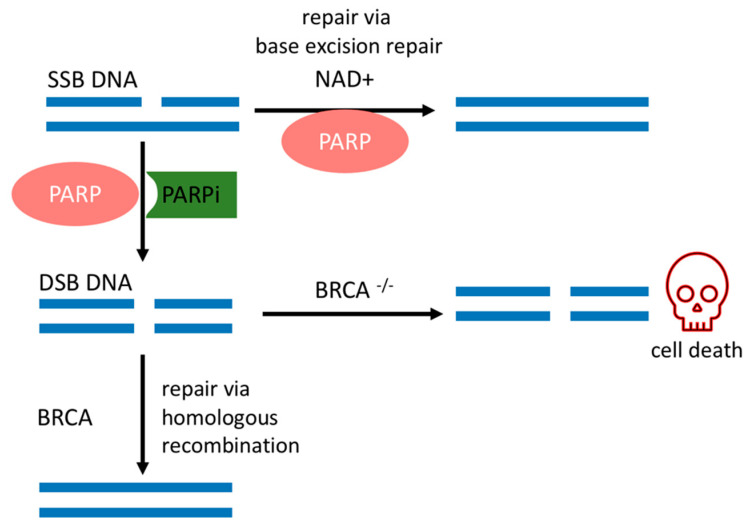
Synthetic lethality. PARPi mechanism of action and relevant DNA repair pathways involved in BRCA mutated cells. SSBs: single-strand break; DSBs: double-strand break; PARP: poly(ADP-ribose) polymerase; PARPi: PARP inhibitor; NAD+: nicotinamide adenine dinucleotide; BRCA: breast cancer gene.

SSBs occur endogenously [20] and can evolve into double-strand breaks (DSBs) during DNA replication, which is the most deleterious DNA insult. In turn, DSBs are repaired by a complex network of proteins that belong to HR. BRCA1 and BRCA2 work at different stages of HR [15] and are required for a fool-proof repair of DNA DSBs through the HR pathway [12]. BRCA1 is a protein that functions in both checkpoint activation and DNA repair. Differently, BRCA2 mediates the core mechanism of HR [21]. HR happens during the S and G2 phases of the cell cycle due to the requirement for a sister template to promote DSB repair [22]. If there is a deleterious mutation of the BRCA1/2 genes, DSB will be fixed by the nonhomologous end-joining (NHEJ) pathway, which is an error-prone pathway. Therefore, BRCA1/2 mutated cancer cells are heavily reliant on a DDR repair pathway that will promptly fix SSBs before they become the deleterious DSBs [18]. Conversely, if PARP activity is inhibited in BRCA-deficient cancer cells, genomic aberrations accumulate beyond a bearable threshold and cause cancer cells to die by synthetic lethality, as depicted in Figure 1 [6,7,23].

The sensitivity of BRCA mutated cancer cells to PARP1 inhibition has brought this enzyme to the forefront of therapeutic interest, and multiple potent candidate molecules have been and continue to be developed [7,8]. All PARPis approved for clinical use, or tested in clinical trials, consist of small synthetic molecules that interact with the NAD+ binding domain. Each PARPi presents a unique pharmacokinetic profile, efficacy [24], and cytotoxicity. Cytotoxicity is dependent on two concurrent mechanisms, namely (1) catalytic inhibition of PAR polymer formation and (2) trapping of PARP1 onto the DNA lesion, forming a complex of PARP1–PARPi–DNA [6]. Their trapping potency is the most relevant factor that leads to synthetic lethality [25], due to the collapse of the replication fork when it encounters the trapped PARP1–PARPi–DNA complexes where deleterious DSBs are formed and cause further cell cytotoxicity [25,26]. This mechanism surpasses the effects of killing cancer cells of only unrepaired SSBs due to the absence of PARP1 in the cell [25].

## 3. GBM: Brief Overview

GBM is a highly aggressive primary brain tumor that commonly acquires resistance to standard therapies [23]. This malignant neoplasia shows a widespread invasive behavior and causes progressive destruction of brain tissues, leading to death [23]. Despite advances in GBM treatment, the median survival time of patients diagnosed with GBM is 14.5 months [1,2,3]. Standard treatment protocols include surgical debulking when feasible, followed by radiotherapy and/or chemotherapy with alkylating agents such as temozolomide, followed by adjuvant use of the same chemotherapeutic [27]. Despite this, fewer than 2% of patients currently survive longer than 5 years after diagnosis [28]. As expected, all current treatment options for GBM face limitations. Surgical resection is challenging due to the infiltrative nature of GBM and the lack of identifiable tumor margins [29]. Moreover, invasive surgery may require withdrawal of adjuvant therapies, thereby reducing treatment options [30]. For radiotherapy, the hypoxic areas of GBMs lead to subtherapeutic concentration of reactive oxygen species, which are responsible for cell toxicity, thus reducing treatment efficacy [31]. In the case of alkylating agents, despite the fact that temozolomide can cross the blood–brain barrier [32], tumor resistance is frequently developed, resulting in GBM regrowth [33].

With a better understanding of GBM biology, novel pharmacological strategies are being developed, most of which target specific molecular pathways, as reviewed elsewhere [34,35]. For example, epidermal growth factor receptor (EGFR), a transmembrane protein, is overexpressed or mutated in 40% of GBM and confers proliferative capabilities to cancerous cells [1]. However, none of the EGFR-targeting drugs showed significant efficacy in patient treatment [36]. Another important GBM treatment approach is the anti-vascular endothelial growth factor antibody bevacizumab with antiangiogenic properties; it received accelerated approval from the Food and Drug Administration (FDA) in 2009 as a single agent for progressive GBM following prior therapy [37]. Unfortunately, further trials and meta-analyses demonstrated that bevacizumab does not improve overall survival for patients newly diagnosed with GBM [38]. New antiglioma therapies are critically needed as GBM remains an incurable disease and PARPis are valuable candidates.

Mounting evidence supports that PARP1 plays a central role in GBM biology. Bartkova et al. demonstrated that the DDR machinery is constitutively activated in GBM tissue even before any exposure to chemotherapy or radiotherapy [39]. Treatment-resistant cell subpopulations, sometimes dubbed GBM stem-like cells (GSCs), present a primed DDR machinery proficient at responding to any DNA insults, including those caused by some conventional anticancer therapies [40,41]. The DDR efficacy of GSCs is linked to the intrinsic oxidative and replicative stress experienced by these cells [40,42]. PARP1 is overactive in GSCs [42] as a response to high levels of nicked DNA [39]. Accordingly, PARP1 expression at the protein and RNA level correlated positively with increasing tumor grade and poorer overall survival compared to reference samples [43]. When inhibitors against components of the DDR machinery, such as PARPis, are employed in GSCs in in vitro experiments, sensitivity to radio- and chemotherapy is rescued, demonstrating the central role of DDR in GBM [40,44].

Among those molecules approved by regulatory agencies and new molecules, three PARPis have undoubtedly demonstrated the capacity to accumulate onto the GBM tissue, beyond plasmatic concentrations, in preclinical models and are being tested clinically, namely veliparib [45,46], niraparib [47], and pamiparib [48]. However, knowledge of biomarkers of the DDR status in GBM is still incipient, limiting the number of strategies available for monitoring the effect of PARPi treatment, tailoring treatment, or determining which patients would benefit from it.

## 4. BRCAness Biomarkers in GBM

**Figure 2:**
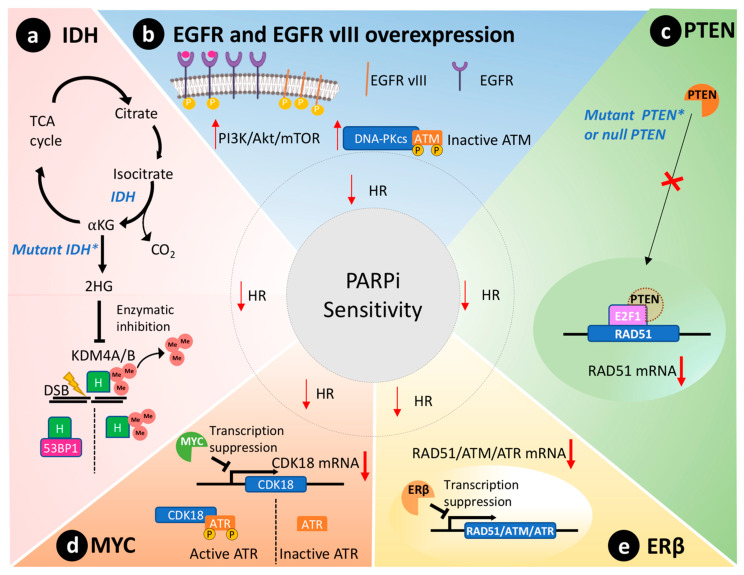
BRCAness phenotype in GBM that leads to impairment of the homologous recombination repair (HR) and PARPi sensitivity. (**a**) The mutated isocitrate dehydrogenase (IDH) enzyme results in the newly acquired ability to produce the oncometabolite 2-hydroxyglutarate (2HG) that inhibits lysine-specific demethylase 4A/B (KDM4A/B), which removes methyl (Me) groups from histones (H). In turn, the methylated histones will hinder the interaction of P53 binding protein 1 (53BP1) with double-strand breaks (DSB) and HR repair. (**b**) The overexpression of epidermal growth factor receptor (EGFR) or the constitutively active EGFR variant III (EGFR vIII) will lead to activation of the PI3K/Akt/mTOR pathway and consequently the activation of DNA-dependent protein kinase catalytic subunit (DNA-PKcs) that will inactivate ATM through phosphorylation. (**c**) PTEN transported to the nucleus induces the transcription of RAD51, an important component of the HR. In GBMs where PTEN is mutated or null, the transcription of RAD51 is reduced, and the HR is impaired. (**d**) MYC protooncogene directly suppresses the expression of CDK18 through interaction with its promoter that is responsible for activating the ATR through phosphorylation. ATR is an important component of HR. (**e**) ERβ is a cytoplasmatic receptor that suppresses the transcription of different components of the HR, such as RAD51, ATM, and ATR. IDH: isocitrate dehydrogenase; TCA: tricarboxylic acid cycle; KG: alpha-ketoglutarate; 2HG: 2-hydroxyglutarate; KDM4A/B: lysine-specific demethylase 4A/B; H: histone; EGFR: epidermal growth factor receptor; EGFR vIII: EGFR variant III; DNA-PKcs: DNA-dependent protein kinase catalytic subunit; ATM: ataxia telangiectasia mutated; PTEN: phosphatase and tensin homolog; EF2: transcription factor 2; ATR: ataxia telangiectasia and Rad3-related; ERβ: estrogen receptor beta.

Biomarkers that reveal the BRCAness status of GBM may predict the outcome for a PARPi administered as a single agent or combined with other therapies [49]. We reviewed data from the most promising candidate biomarkers of BRCAness in GBM, namely mutate isocitrate dehydrogenases 1 and 2 (IDH1/2), EGFR mutated variant (EGFR vIII), phosphatase and tensin homolog (PTEN), MYC oncogene, and the estrogen receptors beta (ERβ). Figure 2 schematically represents the molecular pathways where these biomarkers are involved in ultimately impairing HR.

### 4.1. IDH-1/2

Missense mutations of IDH1/2 genes disrupt HR and affect 9% of GBM tumors [50]. These genes code for enzymes of the citric acid cycle that catalyze the conversion of isocitrate to alpha-ketoglutarate, as depicted in Figure 2a [51]. In contrast, mutated IDH enzymes will convert alpha-ketoglutarate to 2-hydroxyglutarate, an oncometabolite that inhibits lysine-specific demethylase 4A/B (KDM4A/B) [52]. Both enzymes remove histone trimethylation, allowing DDR proteins, such as 53BP1 (P53 binding protein1), to access and repair DSB [53]. Notably, preclinical studies reveal that IDH1/2 mutations predict susceptibility to PARPi therapy [52,54]. Taken together, IDH mutations are expected to cause a BRCAness phenotype through disruption of HR and lead to sensitivity to PARPi therapy in GBM cells [54].

Another impact of IDH1 mutation that modulates PARPi sensitivity is related to alpha-thalassemia/mental retardation syndrome X-linked (ATRX) gene, commonly mutated in lower-grade astrocytoma and secondary GBM [55]. Mutant IDH1 cooperates with mutation/loss of ATRX, driving cells to rely on the alternative lengthening of telomere (ALT) pathway [56]. Preclinical ALT-dependent glioma models were more sensitive to PARPi trapping than ATRX wild type. This sensitivity was attributed to a new mechanism, not dependent on BRCAness [57]. However, other studies indicate that ATRX loss in gliomas can halt HR, inducing PARPi sensitivity [58,59]. Further studies are warranted.

At the time of writing, several clinical trials are ongoing using PARPi in patients presenting with mutated IDH1/2, and the results are awaited eagerly. Two phase I/II clinical trials (NCT03749187 and NCT03914742) will test pamiparib associated with temozolomide for patients that present newly diagnosed or recurrent high-grade glioma with IDH1/2 mutation. Utilizing an inclusion criterion of IDH gene mutation, veliparib is being tested in combination with conventional therapies in a phase II clinical trial (NCT03581292) (Table 1). Currently, olaparib is the only PARPi being tested as a monotherapy in recurrent or refractory malignant gliomas in patients that present confirmed IDH1/2 mutations (Table 1). These clinical trials are at phase II (NCT03212274 andNCT03561870). The later showed that the single-arm trial did not meet its primary endpoint, which was progression-free survival for a six-month period in 45 percent of the 35 enrolled patients. Median progression-free survival was 2.3 months [60].

### 4.2. EGFR vIII

Additionally, the commonly mutated EGFR variant III (EGFR vIII) also modulates DDR in GBM and increases the tumor dependency on PARP1 activity, as represented in Figure 2b [1]. EGFR vIII is a constitutively active mutant that preferentially induces the proliferative phenotype through the activation of the PI3K/Akt/mTOR pathway [61,62]. In turn, this pathway activates the DNA-dependent protein kinase catalytic subunit (DNA-PKcs) [36] that has the capacity to phosphorylate specific sites of ataxia telangiectasia mutated (ATM) protein, thereby deactivating it [63]. ATM is a crucial kinase of HR that triggers checkpoint signaling upon DNA injuries and recruits the machinery responsible for DSB repair. In addition, DNA-PKcs triggers DSB repair through NHEJ preferentially over HR [22,64]. As discussed earlier, NHEJ is an error-prone DNA repair pathway: these cells are highly dependent on PARP1 to avoid death, thus remaining responsive to PARPi therapy [65].

### 4.3. PTEN

PTEN is silenced in 36% of GBMs [23]. Primarily, PTEN regulates the PI3K pathway, suppressing and having an important role in genomic integrity. Shen et al. demonstrated that PTEN through the transcription factor E2F1 transactivates the RAD51 gene promotor, ultimately controlling its gene transcription [66]. Cancer cells without functional nuclear PTEN, or lacking PTEN expression altogether, exhibit reduced RAD51 expression that leads to HR defects [67]. Figure 2c schematically represents this molecular scenario. RAD51 is required to physically link homologous DNA molecules and a processed DNA break to catalyze the DNA strand exchange with an undamaged sister chromatid or homologous chromosome. Cancer cells lacking PTEN present less capacity to repair DNA insults caused by irradiation and are susceptible to PARPi therapy to the same extent as cancer cells presenting BRCA1/2 mutations [67]. Concurrently, PTEN-deficient glioma cells also presented an impaired HR and increased susceptibility towards PARPi therapy [68]. Lin and collaborators described that an orthotopic murine model of PTEN-deficient GBM was more susceptible to the association of veliparib and temozolomide than the control group with wild-type PTEN [69].

A clinical trial using olaparib in combination with temozolomide (NCT01390571) in relapsed GBM patients investigated whether PTEN expression and mutation would result in a different response to treatment. However, this phase I clinical trial was withdrawn due to severe myelosuppression, and PTEN status was not disclosed [70] (Table 1). Further investigation is warranted to elucidate the responsiveness of PARPi treatment in GBM with PTEN deficiency.

### 4.4. MYC

The MYC oncogene family encodes a set of nuclear phosphoprotein transcriptional factors that play key roles in cell cycle progression, apoptosis, cellular differentiation, and metabolism [71]. These genes were found amplified in a subset of GBMs by Hui et al. by genomic microarray analysis [72]. Increased overexpression/amplification of MYC was linked to increased sensitivity to PARPi therapy in patient-derived GSCs [13]. Cancer cells that overexpressed MYC also had a reduction in HR capacity and thus an increased susceptibility to PARPi [13]. Mechanistically, in GSCs, MYC downregulates CDK18, which in turn phosphorylates and consequently activates ataxia telangiectasia and Rad3-related (ATR) kinase, a key regulator of HR, thus conferring susceptibility to PARPi (Figure 2d) [13].

### 4.5. ERβ

Estrogen receptors are proteins present in the cytoplasm and nucleus of cells where they modulate gene expression when they form a complex with estrogen hormone [73]. These receptors play a tumor-suppressive role in GBM models [74,75]. In agreement, aggressive GBM clinical samples showed reduced presence of ERβ in the nucleus compared to less aggressive gliomas and the normal brain tissue [76,77]. Additionally, the use of ERβ agonists increased the sensitivity of GBM to temozolomide treatment in preclinical models [75]. Transcriptome analysis of GBM cells overexpressing ERβ revealed mRNAs related to HR and DDR were downregulated, including RAD51, ATM, and ATR (Figure 2e) [78]. Hence, ERβ expression could be used as a biomarker to predict PARPi susceptibility in GBMs due to its role in modulating the expression of important DDR effectors and associated BRCAness phenotype.

## 5. Clinical Trials and Biomarkers

Considering clinical trials registered in the clinicaltrials.gov database, oncological studies with PARPis encompass more than 100 completed or ongoing ones and are extensively reviewed elsewhere [24]. Olaparib was the first-in-class PARPi approved by the FDA for the treatment of ovarian [79], breast [80], prostate [81], and pancreatic cancer [82] in patients that presented BRCA1 or BRCA2 germline mutations. Subsequently, rucaparib and niraparib [79] were approved for high-grade ovarian cancer, while talazoparib received approval for metastatic breast cancer [79]. Generally, patients with pathological BRCA mutations present the best benefit from PARPi therapy, but clinical studies demonstrated the efficacy of PARPis against cancers presenting defects in HR due to other genes, such as ATM and partner and localizer of BRCA2 (PALB2). As a result, olaparib was approved (May 2020) as a monotherapy for metastatic castration-resistant prostate cancer, for carriers of deleterious mutations in ATM and PALB2 [81,83]. Rucaparib increased overall survival of pancreatic cancer patients carriers of PALB2 in a phase II trial, hence potentially broadening the population that could be benefited by PARPi treatment [84].

PARPi therapies are also evaluated in clinical trials in combination with a myriad of other treatments (reviewed in [27,81,85]). Encouraging results were observed when antiangiogenic agents were combined with PARPis for ovarian cancer treatment [86,87]. Immune checkpoint inhibitors (ICIs), associated with different PARPis, are being tested in clinical trials for treatment of different solid cancers with encouraging results for triple-negative breast cancer patients [85]. The rationale behind this combination is based on the capacity of PARPi to enhance processes such as neoantigen production, antigen presentation, promotion of tumor-infiltrating lymphocytes, and upregulation of programmed cell death ligand 1.

For brain tumors, veliparib is the most tested PARPi. This molecule has been studied in four different phase I trials and in two phase I/II trials where it was combined with temozolomide and/or radiotherapy in all cases. The majority of those clinical trials are now completed, and their results are summarized below.

Chronologically, NCT00649207, completed in 2013, was the first trial to examine whether veliparib could potentiate conventional treatment for brain metastases. The combined treatment produced more favorable median overall survival when compared to historical controls [88]. These results lead to a global phase II trial (NCT01657799) that assessed the effect of veliparib, combined or not with whole-brain radiation therapy (placebo + WBRT). Six years from the start of the study, the final results were published, showing no difference in overall survival between the treatment arms [89].

In 2009, two clinical trials were carried out combining temozolomide with veliparib in pediatric patients (NCT00994071, NCT00946335) [90]. Due to hematologic toxicities, the recommended phase II dose for veliparib was 25 mg/m^2^, and that for temozolomide was 125 mg/m^2^, which is lower than that used for other malignancies [91]. Based on these results, another clinical trial (NCT01514201) [92] was carried out in pediatric patients newly diagnosed with diffuse intrinsic pontine glioma in an attempt to evaluate whether treatment-naïve patients would present less toxicity with higher doses of temozolomide associated with veliparib. In this study, patients tolerated well the maintenance treatment regimen of veliparib (25 mg/m^2^ twice a day) and temozolomide (135 mg/m^2^ daily for 5 days every 28 days) after receiving radiation therapy. The treatment was well tolerated but did not improve overall survival when compared to contemporary historical control groups [92].

Another clinical trial was carried out in adult patients with newly diagnosed GBM (NCT00770471). One of the primary end goals was to determine the maximum tolerated dose of veliparib when administered in combination with radiotherapy and temozolomide. Unfortunately, this study was halted due to increased hematological toxicity in patients receiving 10 mg veliparib twice a day during 42 days and 75 mg/m^2^ of temozolomide daily [93]. Another complete clinical trial using veliparib in brain tumors tried to define its maximum tolerated dose in combination with temozolomide in adults with recurrent GBM (NCT01026493). The combined regimen (temozolomide/veliparib) was tested using two schedules of either 5 or 21 days in a 28-day cycle, which followed two different dose protocols: 40 mg twice daily of veliparib with 75 mg/m^2^ temozolomide, using the 21/28 day schedule, and combined veliparib (40 mg) with temozolomide, using the 5/28 day temozolomide at 150–200 mg/m^2^. Final analysis demonstrated that the treatment did not result in an increased progression-free survival rate when compared to the control group [94]. At the time of writing, there are two active clinical trials, a phase II (NCT03581292) and a phase II/III (NCT02152982), that compare conventional therapy with or without veliparib for newly diagnosed GBM patients; different from the previously mentioned clinical trials of this section, NCT02152982 is systematically assessing the levels of *O*-6-methylguanine-DNA methyltransferase (*O*-MGMT) enzyme as a biomarker of susceptibility to this PARPi [95] (Table 1). Mechanistically, temozolomide induces different types of DNA lesions, and some of them require the repair of PARP1 and *O*-MGMT enzyme [96,97]. Hence, the pharmacological inhibition of PARP1 activity in GBM that presents reduced MGMT expression will result in persistence of lethal DNA lesions and subsequent enhancement of temozolomide cytotoxicity [33]. In fact, epigenetic changes in the MGMT promoter that suppress gene expression are commonly present in GBM and represent a favorable prognostic biomarker for alkylating chemotherapy [98]. Accordingly, preclinical studies demonstrated that PARPi potentiates temozolomide therapeutic effects in low MGMT-expressing tumors [45,99].

In an attempt to sensitize GBM to conventional therapies and overcome resistance mechanisms, other PARPis are being studied. Pamiparib in combination with temozolomide and/or radiotherapy is currently being tested in phase I (NCT03749187) and I/II (NCT03914742 and NCT03150862) clinical trials in GBM patients [9]. Niraparib was tested with temozolomide for safety and efficacy in a phase I trial (NCT01294735), but no results were posted in the clinicaltrials.gov database after completion of the trial in 2012. This PARPi will be tested in a phase II trial (NCT04221503) associated with tumor treating field technology, a treatment expected to disrupt cancer cell migration capabilities and invasiveness through the application of a mild electric field [100] (Table 1).

## 6. Discussion and Conclusions

GBMs are the most aggressive brain cancer, and they are known for genomic abnormalities and resistance to conventional therapies. A better understanding of the cancer biology of GBM has brought into attention the DDR cascade with the central role of PARP1 as a therapeutic target, opening up the opportunity for the use of PARPis in GBM therapy. This resulted in the continuous FDA approval of protocols using PARPis for a growing number of cancer types. Based on preclinical data, clinical trials are evaluating PARPis for the treatment of GBM patients. However, identification of consistent biomarkers that would allow predicting therapy response and elucidation of mechanisms of resistance to these drugs have so far failed.

Since PARP1 enzyme is the target of PARPi molecules, one important feature that must be assessed is its presence and the capacity of these drugs to engage with the enzyme throughout the treatment [43]. Currently, PARP1 levels are determined by immunohistochemistry in biopsy samples from GBM patients [43,101], but the known genetic and morphological heterogeneity of this cancer type limits this strategy as a diagnostic tool [1]. Furthermore, the engagement between PARP1 and its inhibitor requires that the PARPi molecules reach the cell nucleus and remain there to exert their therapeutic effect [102]. Therefore, a crucial characteristic of any PARPi is the capacity to accumulate in GBM tissue [32], evading the molecular efflux pumps [103].

Recent studies have shown that mechanisms of resistance to PARP inhibition are complex and multifaceted, including PARP1 mutations [104], CDK18-mediated resistance [13], and others [105]. Knowledge about these molecular processes and the heterogeneous genetic nature of GBM might explain why it is not possible to recapitulate what is observed in preclinical models in patients. Nonetheless, a growing number of clinical trials are now investigating biomarkers for PARPi sensitivity, and their results are awaited eagerly. Possibly, toxicities observed in some of the previous trials could be overcome with the use of more potent and more specific PARPis [25,106]. Ultimately, in future trials, with the advancement of biomarker research and the systematic use of ‘omics’ technology, a companion diagnostic for GBM should be developed, as has already been done for other cancer types. This will allow a better selection of patients and an optimal combination of targeted therapies.

## Figures and Tables

**Table 1:** Summary of clinical trials using PARPi that have used biomarkers for prediction of PARPi sensitivity.

Drug/Company	Study Start	Associated Therapy	Clinical Trial	Molecular Biomarker	Status	Cancer Type
Olaparib, OR Lynparza, OR (AZD2281) KuDOS/Astra-Zeneca	Mar 2018	monotherapy	NCT03212274 Phase II	mutated IDH1/2	recruiting	glioblastoma
	Jun 2018	monotherapy	NCT03561870 Phase II	mutated IDH1/2	active, not recruiting	recurrent high-grade glioma
	Jul 2011	temozolomide	NCT01390571 Phase I	DNA repair genes, PTEN, MGMT methylation status, mismatch repair	completed	relapsed GBM
Veliparib OR (ABT888) Abbvie	Dec 2018	radiation therapy and temozolomide	NCT03581292 Phase II	DDR markers	recruiting	glioma without H3 K27M OR BRAFV600E mutations
	Dec 2014	temozolomide	NCT02152982 Phase II and III	mutated IDH1/2 MGMT	active, not recruiting	newly diagnosed GBM
	Jul 2009	temozolomide	NCT00946335 Phase I	MGMT, mismatch, DNA repair pathways	completed	pediatric gliomas
Niraparib OR, Zejula, OR (MK4827) Merk/Tesaro	Dec 2019	tumor treating field	NCT04221503 Phase II	methylation status of MGMT	recruiting	recurrent GBM
Pamiparib OR (BGB290) Beigene	Apr 2019	temozolomide	NCT03749187 Phase I	mutated IDH1/2 and others	recruiting	newly diagnosed glioma grade II OR III
	Jan 2020	temozolomide	NCT03914742 Phase I and II	mutated IDH1/2	recruiting	recurrent GBM
	Aug 2017	radiation therapy and/or temozolomide	NCT03150862 Phase I and II	MGMT Methylation	active, not recruiting	newly diagnosed or recurrent/refractory glioblastoma
